# Urocortin, a CRF-like peptide, restores key indicators of damage in the substantia nigra in a neuroinflammatory model of Parkinson's disease

**DOI:** 10.1186/1742-2094-4-19

**Published:** 2007-07-21

**Authors:** Amjad Abuirmeileh, Alexander Harkavyi, Rebecca Lever, Christopher S Biggs, Peter S Whitton

**Affiliations:** 1 Department of Pharmacology, The School of Pharmacy, 29–39 Brunswick Square, London WC1N 1AX, UK; 2 School of Biosciences, University of Westminster, 115 New Cavendish Street, London W1W 6UW, UK

## Abstract

We have recently observed that the corticotrophin releasing hormone (CRF) related peptide urocortin (UCN) reverses key features of nigrostriatal damage in the hemiparkinsonian 6-hydroxydopamine lesioned rat. Here we have studied whether similar effects are also evident in the lipopolysaccaride (LPS) neuroinflammatory paradigm of Parkinson's disease (PD). To do this we have measured restoration of normal motor behaviour, retention of nigral dopamine (DA) and also tyrosine hydroxylase (TH) activity. Fourteen days following intranigral injections of LPS and UCN, rats showed only modest circling after DA receptor stimulation with apomorphine, in contrast to those given LPS and vehicle where circling was pronounced. In separate experiments, rats received UCN seven days *following *LPS, and here apomorphine challenge caused near identical circling intensity to those that received LPS and UCN concomitantly. In a similar and consistent manner with the preservation of motor function, UCN 'protected' the nigra from both DA depletion and loss of TH activity, indicating preservation of DA cells. The effects of UCN were antagonised by the non-selective CRF receptor antagonist α-helical CRF and were not replicated by the selective CRF_2 _ligand UCN III. This suggests that UCN is acting via CRF_1 _receptors, which have been shown to be anti-inflammatory in the periphery. Our data therefore indicate that UCN is capable of maintaining adequate nigrostriatal function *in vivo*, via CRF_1 _receptors following a neuro-inflammatory challenge. This has potential therapeutic implications in PD.

## Findings

Parkinson's disease (PD) is largely the result of a degeneration of nigrostriatal neurons. Before the disease presents clinically, death of dopamine (DA) neurons occurs in the substantia nigra pars compacta (SNc) asymptomatically. This has traditionally been ascribed to concurrent apoptotic, excitotoxic and free-radical mediated events [1,2] Recent evidence suggests that both pre- and postnatal neuroinflammation may play a crucial predisposing or causative role in the aetiology of PD [3,4]. Prevention of nigrostriatal neuronal destruction once established, or prior to lesion development, represents an ideal future therapeutic goal in PD. Urocortin (UCN), a corticotrophin releasing hormone (CRF) related peptide has recently been proposed as a cytoprotectant. Evidence for this exists in a range of tissues including neuronal cells [5,6]. Interestingly UCN, acting via CRF_1 _receptors, is anti-inflammatory in the periphery [7]. We have recently observed that UCN arrests the development of Parkinsonian like features in the 6-hyroxydopamine lesioned hemiparkinsonian rat [8]. UCN substantially reverses apomorphine-induced circling, loss of tissue DA, loss of nigral and striatal tyrosine hydroxylase (TH) activity and loss of TH protein levels [8]. Although the 6-OHDA model of PD is well established it has significant physiological limitations. In contrast, lipopolysaccaride (LPS) is an established product of bacterial infection, including relatively common conditions such as bacterial vaginitis. Significantly, evidence suggests that systemic inflammation can predispose or be causative in the genesis of PD [3,4]. Therefore, exposure to conditions leading to neuroinflammation, a condition to which the SNc in particularly susceptible, constitutes a realistic mechanism by which the disease may be initiated. Here we have investigated the potential protective effects of UCN in the LPS paradigm of PD. Additionally, we have studied the effects of the non-selective CRF receptor antagonist α-helical CRF and also urocortin III (UCN III), a selective CRF_2 _agonist [9], on indices of nigral DA neuronal integrity to determine whether effects of UCN are receptor mediated and the likely subtype.

**Figure 1:**
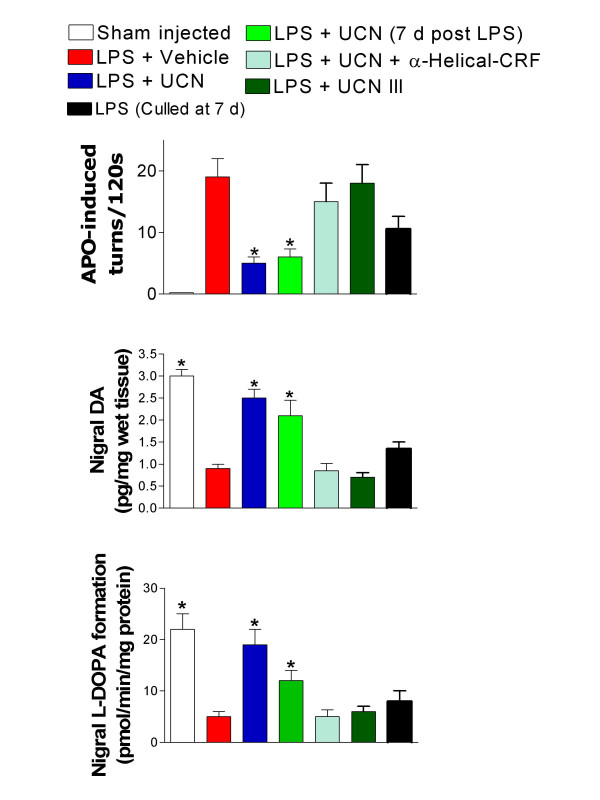
Effect of the CRF-like peptide UCN on indices of nigrostriatal damage induced by intranigral injection of LPS. Data were taken from rats 14 days after injection of LPS and UCN except where indicated (7 d post LPS) in which case UCN was given seven days *after *LPS injection. In some experiments rats were culled seven days after administration of LPS alone to indicate the development of lesion severity at this point. Indices assessed were as follows: upper panel, circling behaviour in response to the DA agonist apomorphine (one way ANOVA F = 7.763, p < 0.001); middle panel, nigral tissue DA concentration (F = 22.77, p < 0.001), lower panel, nigral tissue TH activity (F = 11.47, p < 0.001). **Each group comprised 6–8 rats. **In each case differences between groups were assessed using Bonferonni's multiple comparison test. *p < 0.05 versus groups treated with either LPS and vehicle or LPS and UCN III.

UCN, UCN III, LPS, α-helical CRF and apomorphine were all obtained from Sigma, UK. The latter agent was dissolved in 0.2% w/v ascorbic acid, whilst LPS, α-helical CRF, UCN and UCN III were initially dissolved in water and further diluted in saline. The concentration of UCN chosen is identical to that used in previous investigations [8,10,11]. Apomorphine was injected in a volume of 0.1 ml per 100 g body weight. Experiments were performed in accordance with the Animals (Scientific Procedures) Act, UK (1986). Male Wistar rats (Charles River, UK; 210–240 g) were group housed with food and water *ad libitum*. Animals were anaesthetised, secured in a stereotaxic frame and given injections of LPS (2 μg/2 μl) into SNc (from bregma in mm; A -5.2, L 2.2 and V 8.3). Animals co-treated with UCN, UCN III or α-helical CRF received injections (20 fmols/2 μl) directly into the ipsilateral SNc whereas those not receiving UCN were given vehicle. UCN itself was administered either concomitantly with LPS or *7 days later *(i.e. once lesions were clearly evident; Fig. 1). All intracerebral injections were performed using a stereotaxic frame mounted microsyringe (Hamilton, US) over approximately four minutes (0.5 μl/min.) and the needle left in place for five min post-injection. Fourteen days after toxin administration (seven days in certain experiments, Fig. 1) rats were given apomorphine (0.5 mg/kg, s.c.) and rotations measured 30 min later for 2 min in a circular 'arena', approximately 1 metre in diameter, to estimate lesion severity. Rats were then lightly anaesthetised, brains removed and their substantia nigra dissected on ice. DA was estimated as previously described [12] and TH activity estimated as outlined previously [8]. Data were subjected to one way ANOVA with a *post hoc *Dunnett's test.

Rats treated with LPS and vehicle displayed characteristic intense, 'tight' contraversive circling, following apomorphine (Fig 1) while those co-treated with intracerebral UCN displayed much reduced apomorphine sensitivity. UCN III did not attenuate apomorphine-induced circling in lesioned rats (Fig 1). LPS treatment produced drastic decreases in nigral DA which were substantially attenuated when UCN was injected at the same time as the LPS (Fig. 1) and critically, when UCN was given seven days *following *LPS injection. A similar pattern of reduction was seen in nigral TH activity following LPS, and this was also reversed by UCN either given at the same time and also seven days post LPS injection (Fig. 1). In none of the experiments did UCN III effect any decrease in LPS-induced loss of tissue DA or TH activity. In order to establish whether these apparent reductions in 'Parkinsonian-like' pathology were mediated by CRF receptors we co-administered LPS and UCN with the non-selective CRF receptor antagonist, α-helical CRF. α-helical CRF reversed the 'protective' effects of UCN against LPS-induced loss of DA, TH activity as well as apomorphine-induced circling (Fig. 1). This clearly indicates that CRF receptors mediate the actions of UCN. However, the CRF_2 _selective analogue of UCN, UCN III was without effect. This logically indicates that the protective actions of UCN are mediated by CRF_1 _receptors.

The current findings demonstrate that UCN greatly attenuates the development of PD-like pathology in a recently proposed [3,4] paradigm of the illness (LPS). The validity of this model is being increasingly appreciated as the role of neuroinflammation as a factor in the aetiology of PD is gaining substantive support in patients and animal-models [3,4,13]. The ability to restore these indices of PD-like damage in dopaminergic nigral neurons seven days after administration of LPS is particularly significant. We have determined that at this time point in our models the lesion has become established but is evidently still unstable since degeneration continues to proceed for a least a further seven days [8,14], Fig. 1. This is reasonably analogous to the predicament of PD patients, where degeneration proceeds until the nigrostriatal system is to all intents destroyed as the illness reaches its terminal phase. Current treatments are of limited, and purely symptomatic value, becoming ineffective as the neurodegeneration proceeds. What is clearly required is some treatment strategy which either stabilizes the hostile conditions prevailing within the SNc, or better, effects some degree of neuronal restoration. Our data suggest that UCN may be able to achieve this, probably acting via CRF_1 _sites. The observations with UCN suggest that under neuroinflammatory conditions it is able to elicit a functional recovery in nigrostriatal neurotransmission. We have previously found UCN I to be effective in restoring both striatal TH activity and DA content following either LPS or 6-OHDA-induced lesions [8,14]. Futhermore, we have found that UCN I also reverses loss of extracellular DA in the striatum of freely moving rats (unpublished data). The resulting recovery in nigral DA neurons presumably allows for a restoration of D_1 _and D_2 _receptor balance in the striatum which would logically underlie the recovery in 'normal' motor activity (loss of circling) seen after apomorphine treatment. However, a determination of actual DA receptor population would be required to prove this.

Evidence has shown that UCN protects some neurons via activation of the CRF_1 _subtype [5,6], whilst activation of CRF_2 _sites has been cited as important [10,15]. The possibility that UCN can 'rescue' damaged cells has been postulated in some studies, especially cardiac myocytes [10] and the heart ex vivo [11,16]. We are unclear as to the precise mechanism by which UCN I exerts its protective effect. Our unpublished data indicates that UCN I treatment leads to a preservation or restoration of TH+cells in the SNc. Whether this is the result of cytoprotection, such as might occur due to an anti-inflammatory action, or a stimulation of neurogenesis remains to be determined. One possibility could be that UCN I might reduce the massive astrogliosis which arises in the SNc as a result of LPS toxicity [17]. Additionally, the potential contribution of the SNc relative to the ventral tegmental area in restoring nigrostriatal function is also unclear, although we intend to investigate this.

In summary, our data constitutes the first report of a restoration of key indicators of nigrostriatal damage in a neuroinflammatory model of PD *after *the lesion has become established by a molecule known to have antiinflammatory properties [7]. Although activation of the HPA axis by a CRF agonist might have potentially deleterious side effects, evidence suggests that these may be averted. Thus, CRF and UCN both reduced weight gain in rodents but CRF was much more effective than UCN in this respect and only CRF produced effects consistent with increased sympathetic activity[18]. In order to achieve substantial therapeutic relevance a means by which central CRF_1 _sites can be activated is clearly essential as may be refinement to ensure an appropriate pharmacological response. While UCN is a relatively large molecule with poor blood brain barrier penetration, recently small, lippophillic, CRF_1 _selective antagonists have become available and CRF receptor pharmacology is a rapidly expanding field. As such we consider it highly likely that CRF_1 _agonists will become available offering new possibilities in the study of UCN mediated neuroprotection as well as being of potential therapeutic value in PD.

## Abbreviations

Dopamine (DA), corticotrophin releasing factor (CRF), **hypothalamopituitary-adrenal (HPA)**, lipopolysaccaride (LPS), Parkinson's disease (PD), tyrosine hydroxylase (TH), urocortin (UCN).

## Competing interests

The author(s) declare that they have no competing interests.

## Authors' contributions

AA, AH, CSB and PSW were responsible for the planning and actual experimentation involved in this study. RL contributed to the interpretation of the data and writing of the manuscript.
